# Insomnia, OSA, and Mood Disorders: The Gut Connection

**DOI:** 10.1007/s11920-024-01546-9

**Published:** 2024-10-14

**Authors:** André P. Pacheco, Jonathan Cedernaes, Christian Benedict

**Affiliations:** 1 Department of Research and Innovation, Division of Mental Health and Addiction, Oslo University Hospital, Sognsvannsveien 21, Oslo, 0372 Norway; 2 Institute of Clinical Medicine, Faculty of Medicine, University of Oslo, Oslo, Norway; 3 Department of Medical Sciences, Uppsala University, Uppsala, Sweden; 4 Department of Medical Cell Biology, Uppsala University, Uppsala, Sweden; 5 Department of Pharmaceutical Biosciences, Uppsala University, Husargatan 3, Uppsala, 751 24 Sweden

**Keywords:** Gut microbiome, Sleep disorders, Insomnia, Obstructive sleep apnea, Mood disorders

## Abstract

**Purpose of Review:**

With the growing body of research examining the link between sleep disorders, including insomnia and obstructive sleep apnea (OSA), and the gut microbiome, this review seeks to offer a thorough overview of the most significant findings in this emerging field.

**Recent Findings:**

Current evidence suggests a complex association between imbalances in the gut microbiome, insomnia, and OSA, with potential reciprocal interactions that may influence each other. Notably, specific gut microbiome species, whether over- or under-abundant, have been associated with variation in both sleep and mood in patients diagnosed with, e.g., major depressive disorder or bipolar disorder.

**Summary:**

Further studies are needed to explore the potential of targeting the gut microbiome as a therapeutic approach for insomnia and its possible effects on mood. The variability in current scientific literature highlights the importance of establishing standardized research methodologies.

## Introduction

The growing recognition of the link between gut microbial richness and sleep efficiency, such as the observed association in young adults [1], has sparked increased interest in the gut microbiome as a critical area of study within sleep science and medicine. As a diverse community of microorganisms residing in the gastrointestinal tract, the gut microbiome is now seen as a potential contributor to the development and worsening of sleep disorders [2–4].

From a public health standpoint, the two most pressing and most prevalent sleep disorders are chronic insomnia and obstructive sleep apnea (OSA). Chronic insomnia affects approximately 10% of the population in industrial societies [5], and is characterized by persistent sleep disturbances despite adequate sleep opportunity, with difficulties to initiate or maintain sleep, or awakening too early, combined with adverse daytime consequences [6]. OSA, affecting nearly 1 billion people worldwide [7], is defined by repeated episodes of partial or complete upper airway obstruction during sleep. Consequently, airflow is intermittently reduced or absent, which results in disrupted and unrefreshing sleep, leading to common symptoms experienced by patients with OSA, such as daytime sleepiness [8].

Given the rapid increase in studies exploring the relationship between the gut microbiome and both insomnia and OSA [2–4], this review aims to provide a concise yet comprehensive overview of the key findings in this area. We begin with an initial section defining essential terminology before critically synthesizing evidence that shows how individuals with insomnia and OSA exhibit distinct gut microbiome profiles compared to healthy controls. In light of the established link between insomnia and OSA, and mood disorders such as depression [9, 10], we further investigate whether the gut microbial characteristics of these sleep disorders overlap with those observed in common mood disorders.

Moreover, insomnia can affect biological systems in the body, such as the immune system [11], thereby likely creating selective pressures on systems like the gut microbiome [12]. Adaptive changes in the gut microbiome, with implications for bacterial functions, such as the production of metabolites that can affect the host, and the diversity of the microbiome, may in turn influence the host’s sleep and behavior. To explore these complexities, we also review potential underlying mechanisms that could explain this bidirectional relationship. Finally, we examine recent evidence indicating that associations between sleep disruption and the gut microbiome can vary considerably across studies. Therefore, we discuss several factors that might contribute to these inconsistencies in the scientific literature [13, 14].

By synthesizing this information, we aim to provide a comprehensive overview of the relationship between disruptions in the gut microbiome and the pathology of insomnia and OSA, as well as potential overlaps with mood disorders. Additionally, we discuss the implications of these findings for treatment strategies and identify potential directions for future research in this evolving field.

## Deciphering the Microbiome

An understanding of the association between insomnia, OSA, and the gut microbiome requires familiarity with several key terms and concepts. The gut microbiome comprises the entirety of the microorganisms that reside in the gastrointestinal tract, such as bacteria, viruses and fungi, which constitute the microbiota, and their genetic material. For practical reasons, research on human health and the gut microbiota has mainly focused on gut bacteria found in the lower gastrointestinal tract, as assessed by fecal microbiome analyses. Gut bacteria are classified hierarchically into phylum, class, order, family, genus, and species. Recent changes in the classification of major gut phyla have introduced the terms Actinomycetota, Bacillota, Bacteroidota, and Pseudomonadota, which were previously known as Actinobacteria, Firmicutes, Bacteroidetes, and Proteobacteria, respectively [15]. This change of classification is important to keep in mind when interpreting past and future research. To account for differences in phylogenetic developments not covered in previous literature, the legacy terms used in their respective publications will be retained in this review.

Several parameters describe the gut microbial landscape. Alpha diversity, generally associated with better overall health when high, refers to the variety of species within a single microbiome community and encompasses two main aspects: richness and evenness [16, 17]. Richness indicates the number of different species present, while evenness reflects how evenly these species are distributed [18–20]. Another important parameter is beta diversity, which examines differences in microbial communities across similar environments, such as comparing stool samples from different individuals [21]. For instance, even if two individuals follow the same diet, their gut bacterial compositions can differ significantly.

Lastly, gut dysbiosis (an imbalance in the microbial community), can contribute to various health issues and disease progression, making it a key target for therapeutic intervention. To correct such imbalances, therapeutic strategies often focus on modifying the gut microbiome through the use of prebiotics and probiotics. Prebiotics stimulate the growth of beneficial bacteria by supplying essential nutrients, while probiotics involve the direct supplementation of specific bacterial strains to restore or enhance a healthy microbial balance.

## Microbial Signatures and Therapeutic Prospects in Insomnia

When reviewing results from studies investigating the association between insomnia and the gut microbiome, several challenges arise. These include differences in the definition of insomnia across studies and uncertainty about the chronicity and severity of the condition. Such variations present significant obstacles to synthesizing current evidence on the relationship between this sleep disorder and the gut microbiome.

Despite these limitations, several studies have examined the link between insomnia and the gut microbiome in humans. For instance, a case-control study found that the gut microbiota in 58 patients with insomnia compared with healthy controls was characterized by lower microbial richness and diversity, depletion of anaerobes and short-chain fatty acid (SCFA)-producing bacteria [22]. These findings are consistent with other results [23, 24], including a larger study involving several thousand donors of fecal samples, which also observed reduced microbial diversity among subgroups of patients with varying levels of chronic insomnia [23].

When considering disorder chronicity, in the aforementioned case-control study [22], individuals with acute insomnia (*n* = 20), defined as lasting more than one week but less than three months, exhibited a lower relative abundance of Firmicutes compared with healthy controls (*n* = 38). Highlighting how chronic insomnia may differently affect the gut microbiome, the same study observed that patients with chronic insomnia (*n* = 38) had an increased abundance of the phylum Actinobacteria, along with overrepresentation of the genera *Blautia* and *Eubacterium hallii*, and lower abundance of the genera *Faecalibacterium*, *Prevotella*, and *Roseburia* [22]. Complementing these observations, a separate study of 17 patients with chronic insomnia found an increased abundance of the genus *Eggerthella* [25]. Additionally, another study involving 40 patients with insomnia reported an increased relative abundance of *Lactobacillus* and *Streptococcus* [24]. Finally, in two large cohorts involving several thousand single-time fecal samples, *Ruminococcaceae* species were identified as microbial biomarkers of chronic insomnia [23]. Notably, many gut microbial features associated with insomnia, as highlighted in this section, also overlap with those observed in mood disorders, which we will discuss further.

While no published study has yet investigated whether the recommended first-line treatment for chronic insomnia, cognitive behavioral therapy for insomnia [26], can alter the gut microbial landscape to resemble that of healthy controls, recent studies suggest that transplanting fecal microbiota from healthy donors to patients with insomnia may offer promising therapeutic benefits. Specifically, fecal microbiota transplantation (FMT) significantly improved sleep quality and reduced insomnia severity in an interventional prospective study of 33 adults with chronic insomnia [25], as well as in an open-label study involving adults with post-acute Coronavirus disease 2019 syndrome and insomnia [27]. Patients in these studies demonstrated marked improvements in insomnia severity, sleep quality, anxiety, and depression. This was seen alongside higher levels of intestinal bacteria such as *Bifidobacterium* and *Lactobacillus* in one study [25], and *Coprococcus comes* and *Gemmiger formicilis* in the other study [27]. Additionally, FMT recipients achieved greater remission from insomnia compared with the control group [27]. The increase in *Coprococcus* is notable, as these tend to be found at lower levels in certain mood disorders [14].

Taken together, current evidence suggests that the gut microbiome differs between individuals with insomnia and healthy controls. Although findings vary, possibly depending on the duration of the sleep disorder, the prevailing evidence indicates that insomnia is often associated with reduced microbial richness and diversity. Whether these changes in microbiome diversity are a cause or a consequence of insomnia remains unclear. However, interventions aimed at increasing gut microbiome diversity, such as FMT, have been shown to improve sleep [25, 27]. This supports the hypothesis that gut microbiome features associated with insomnia may play a causal role in this sleep disorder.

## How OSA Interacts with the Gut Microbiome

Studying the impact of OSA on the human gut microbiome presents several challenges. OSA frequently occurs alongside other conditions that affect the gut microbiome, such as obesity, type 2 diabetes, hypertension [28, 29], and is strongly associated with gastroesophageal reflux disease [30]. Long-term use of metformin, a common medication used to treat type 2 diabetes, and proton pump inhibitors like omeprazole, used to treat gastroesophageal reflux disease, are known to significantly impact the gut microbiome [31, 32]. Further complicating the issue, OSA varies in severity, typically measured by the apnea-hypopnea index (AHI), which quantifies the average number of apneas and hypopneas per hour during sleep.

Despite these complexities, several studies have investigated how the gut microbiome changes with varying levels of OSA severity. For instance, a study involving 48 participants with varying degrees of OSA found that more severe OSA may be linked to an increased abundance of *Fusobacterium* in the gut [33]. This species is also overrepresented in patients with generalized anxiety disorder [34]. Since a meta-analysis found that anxiety affects about one-third of patients with OSA [35], this could suggest that *Fusobacterium* plays a role in this association.

The most significant findings regarding the impact of OSA on gut microbiome diversity and composition come from the Swedish Cardiopulmonary BioImage Study, which analyzed fecal samples from 3,570 adults. This largest study to date found both lower alpha and beta diversity with increased OSA severity [36]. This indicates that OSA severity is associated with reduced overall microbial diversity and a more uniform microbial community composition among individuals with severe OSA. In contrast, a smaller study involving 19 OSA patients and 20 healthy controls did not observe significant differences in diversity measures between those with and without OSA [37]. This discrepancy highlights the need for large-scale studies to better understand how sleep disorders like OSA affect the gut microbiome.

Changes in the gut microbiome associated with OSA may even begin early in life. For example, two case-control studies involving children with OSA provide evidence for this. One study showed that 16 children aged 2 to 12 years with OSA had decreased microbial diversity compared to healthy peers [38]. Another study involving 43 two-year-olds found that a primary symptom of OSA, snoring, was linked to lower microbial diversity and richness, as well as a higher ratio of Firmicutes to Bacteroidetes [39]. Increased abundance of Firmicutes relative to Bacteroidetes has also been observed in some studies of children with autism spectrum disorder [40]. This is of particular interest given that a significant proportion of children with autism have comorbid OSA [41]. Notably, the pattern of an increased Firmicutes to Bacteroidetes ratio may be more characteristic of pediatric patients with OSA rather than adults. For example, a study involving 93 adults with varying degrees of OSA severity found no correlation with the Firmicutes to Bacteroidetes ratio [42].

Although interventional studies in humans are limited, a recent pilot study involving eight middle-aged adults with moderate-to-severe OSA found no significant changes in the gut microbiome composition after two months of continuous positive airway pressure (CPAP) therapy [43]. This is noteworthy, because CPAP is an effective standard treatment for reducing the frequency of breathing pauses during sleep in patients with OSA. These findings align with earlier research showing no alterations in intestinal dysbiosis following the normalization of oxygen saturation in animal models [44]. Nevertheless, it is important to consider that negative results in these studies may be due to small sample sizes. This could limit the ability to detect potential effects of breathing pauses and associated hypoxia on the human gut microbiome.

## Gut Microbiome: The Link between Mood, Insomnia, and OSA?

Given that up to two-fifths of patients with mood disorders experience sleep disturbances [45], and that mood disorder symptoms are closely linked to disordered sleep [46], an intriguing research question arises: could the overlap in microbial characteristics between mood disorders, insomnia, and OSA indicate shared gut-related mechanisms? While still in its infancy, research has begun to offer some answers to this intriguing question. For instance, the phylum Firmicutes is notably reduced in the fecal samples of patients with major depressive disorder (MDD) [47], a pattern also observed in patients with acute insomnia [22]. A recent systematic review and meta-analysis reported an increased abundance of the phylum Actinobacteria and the genus *Eggerthella*, along with reduced levels of the genus *Faecalibacterium*, in fecal samples of patients with MDD, bipolar disorder or schizophrenia [14]. As previously discussed, these microbial changes are consistent with those seen in insomnia [22, 25]. Moreover, the pattern of the depletion of *Ruminococcaceae*, observed in patients with chronic insomnia [23], has been linked to greater MDD severity [48], although this association is not uniform across all MDD studies [14]. Additionally, the pattern of increased relative abundance of the genera *Lactobacillus* and *Streptococcus*, found in patients with chronic insomnia [24], has been associated with higher symptom severity in individuals with MDD, bipolar disorder, and schizophrenia-spectrum disorders [49].

Notably, a study analyzing stool samples from 36 patients with MDD found that higher abundances of the genera *Dorea*,* Blautia* and *Coprococcus* were associated with better sleep quality, as measured by the Pittsburgh Sleep Quality Index, and lower depression severity, as assessed by the Hamilton Depression Rating Scale (HAM-D) [50]. These genera are known producers of SCFAs [51–53], which have been positively correlated with improved sleep [54–56] and mood [57–59]. Thus, therapies aimed at increasing the abundance of these genera may hold promise for simultaneously improving both sleep and mood in patients with MDD.

A recent meta-analysis found that the majority of studies, though not all, reported a lower abundance of *Faecalibacterium* among patients with MDD or bipolar disorder [14]. This finding is noteworthy as a higher fractional representation of *Faecalibacterium* has been linked to better sleep quality and lower generalized anxiety in 115 patients with bipolar disorder [60]. *Faecalibacterium* is one of the most abundant SCFA-producing bacteria in the gut [61], and its role in SCFA production may contribute to its association with improved sleep and mood regulation.

Targeting the gut microbiome has shown potential in alleviating symptoms of both depression and disturbed sleep. For instance, a study involving 17 patients, 12 of whom had mild depression, demonstrated that FMT from healthy donors resulted in significant improvements in both the HAM-D total score and the sleep subscale score [62]. Similarly, two recent randomized controlled trials involving 110 participants indicated that probiotic supplementation, when used adjunctively to antidepressant medication, can reduce depressive symptoms in MDD and alter gut microbial composition compared to controls [63, 64]. While these studies included sleep-related items as part of the depressive symptom scales, suggesting that improvements in depression may also reflect better sleep, none specifically focused on sleep subscales.

Taken together, these findings underscore the potential of targeting the gut microbiome as a therapeutic approach to alleviate both mood and sleep disorders.

## How Insomnia and OSA May Transform Microbial Landscapes and Vice Versa

**Fig. 1:**
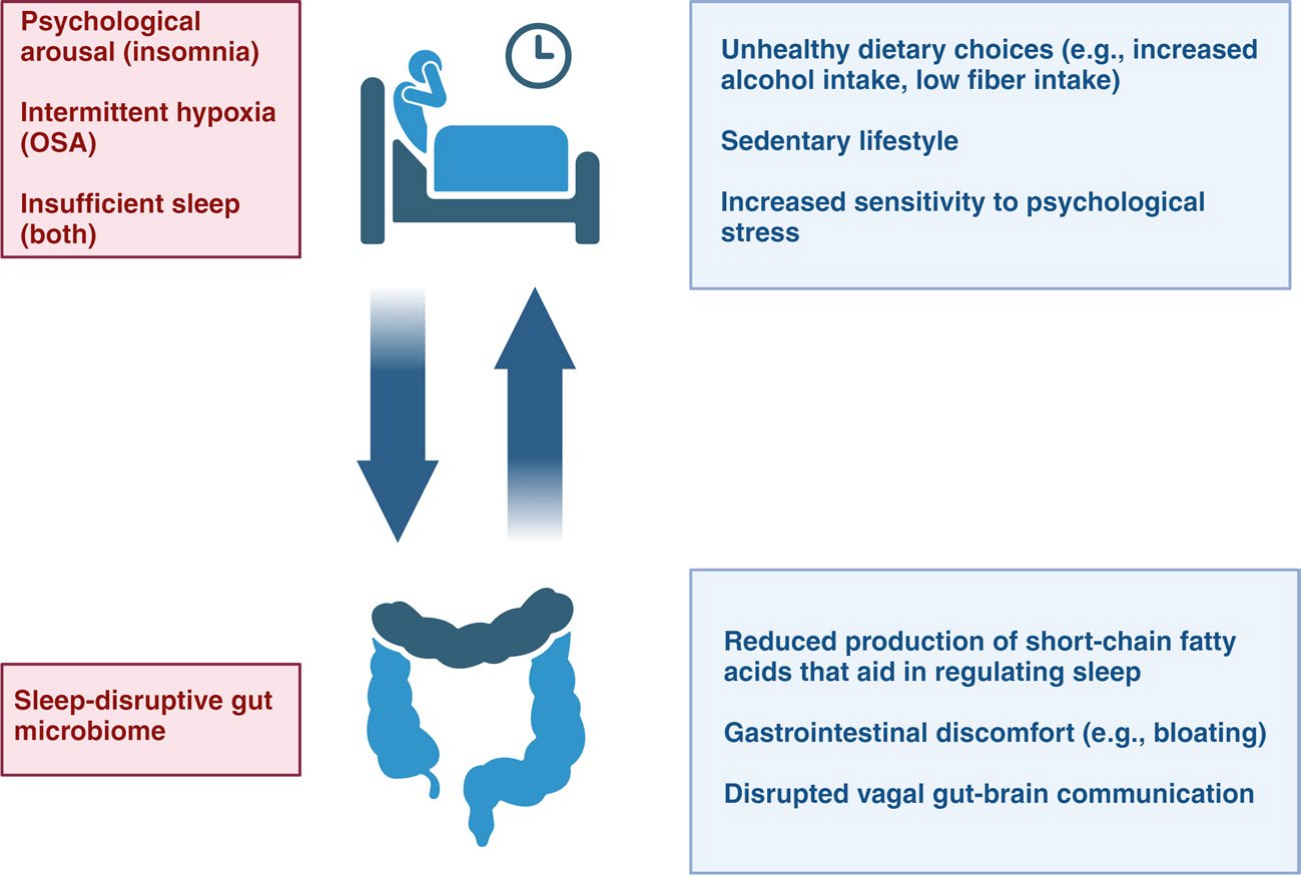
Interplay between gut microbiota disruption and disturbed sleep. Various physiological and psychological factors associated with disturbed sleep, such as poor food choices, are linked to changes in the gut microbiome. An imbalanced gut microbiome, characterized by low microbial diversity and the prevalence of certain microbes, may be associated with further sleep disturbances, potentially contributing to a recurring cycle

Given that most studies exploring the associations between insomnia, OSA, and the gut microbiome are cross-sectional, it remains unclear whether these sleep disorders directly lead to changes in the gut microbiome or if gut microbiome alterations contribute to the development of these conditions. However, as summarized in Fig. 1, evidence suggests a bidirectional relationship.

Insufficient sleep, commonly associated with insomnia or OSA, has been shown to trigger behavioral and metabolic changes that negatively impact gut microbiome diversity and composition [65–70]. These changes include overeating, poor dietary choices, reduced physical activity, and increased sensitivity to psychological stress [71–80]. This supports the idea that differences in gut microbiome profiles between individuals with insomnia and OSA compared to healthy controls, as discussed earlier, may be a consequence of the underlying sleep disorders.

Conversely, there is also evidence suggesting that disruptions in the gut microbiome could play a role in causing sleep disturbances associated with insomnia and OSA. For instance, gut dysbiosis can lead to digestive issues such as abdominal pain, bloating, constipation, and altered bowel movements [81], which can disrupt sleep, especially if they occur at night. Additionally, a reduction in butyrate-producing bacteria, such as *Faecalibacterium prausnitzii*, has been linked to both OSA and insomnia [22, 38]. SCFAs like butyrate influence genes that regulate circadian rhythms and sleep [54–56].

Moreover, the gut microbiota might influence sleep via the vagus nerve, the body’s tenth cranial nerve, which contains both efferent and afferent neurons [82]. Extensive animal research has shown that the gut microbiome can affect central nervous system activity by stimulating vagal afferents through mechanisms such as the release of butyrate, or neurotransmitters such as gamma-aminobutyric acid and serotonin [83]. Notably, evidence from studies on epileptic patients suggests that vagal stimulation can impact both breathing patterns during sleep and sleep quality [84–86].

In summary, while most research on the connections between insomnia, OSA, and the gut microbiome is cross-sectional and suggests a possible bidirectional relationship, the precise mechanisms remain unclear. Insufficient sleep, common in both conditions, is linked to behavioral and metabolic changes that negatively affect gut microbiota. Conversely, gut dysbiosis and reduced production of microbial metabolites, such as SCFAs, which are involved in regulating sleep and circadian rhythms, may exacerbate sleep disturbances. This complex, interdependent relationship requires further investigation.

## Challenges in Studying Sleep and Gut Microbiome Interactions

When studying the associations between sleep or mood disorders and the gut microbiome, several factors must be considered. Key variables include body mass index, dietary habits, medication use (not only antibiotics), biological sex, and the timing of fecal sample collection. Each of these factors can markedly influence the human gut microbiota [32, 87–90]. Additionally, the methods used to assess insomnia and OSA can vary significantly between studies, which often do not adhere to established clinical guidelines and standardized protocols. Such variations encompass the diagnostic criteria, sleep assessment tools, and evaluation techniques used in different studies. For instance, some studies rely on self-reported questionnaires or sleep diaries, while others utilize objective measures such as polysomnography or actigraphy.

The heterogeneity of sleep disorders also complicates comparisons. For example, insomnia is classified into acute, recurrent, or chronic insomnia, with or without co-morbid conditions [6]. Similar individual differences must be considered when studying the gut microbiome in OSA. For instance, OSA may or may not be associated with excessive daytime sleepiness [91], and can coexist with insomnia, a condition referred to as COMISA [92]. It is crucial to apply stringent criteria in studies of OSA, to ensure that control groups are free from the condition, given that many individuals are unaware of their OSA status [91, 93]. Furthermore, single-night sleep apnea screenings can lead to misclassification [94].

Factors that can impact the quality of fecal sampling, including improper or subpar sample collection, storage or transportation, can risk contamination and low-quality samples, issues which can further complicate the comparison of studies. Additionally, differences in sample sizes across studies, the impact of human genetics on the gut microbiome with heritable traits observed in monozygotic twins [95], and ethnic and geographic variations [96] can limit the ability to draw effective comparisons. In summary, achieving a comprehensive understanding of these complex interactions necessitates meticulous attention to methodological variability and careful consideration of these various factors.

## Conclusion

Targeting the gut microbiome offers a potential avenue for the treatment of sleep and mood disorders. To better understand this possibility, there is a need for interventional studies that explore how microbiome modulation might be integrated into clinical practice. Such research will be important for evaluating these approaches, refining treatment protocols, and assessing their impact on patient care.

## Data Availability

No datasets were generated or analysed during the current study.
